# Effect of Mixer Type on Particle Coating by Magnesium Stearate for Friction and Adhesion Modification

**DOI:** 10.3390/pharmaceutics13081211

**Published:** 2021-08-05

**Authors:** Wei Pin Goh, Ana Montoya Sanavia, Mojtaba Ghadiri

**Affiliations:** Faculty of Engineering and Physical Sciences, University of Leeds, Leeds LS2 9JT, UK; w.p.goh@leeds.ac.uk (W.P.G.); pm19ams@leeds.ac.uk (A.M.S.)

**Keywords:** mixing, coating, magnesium stearate, lubricant, Turbula, ProCepT, Mechanofusion, flowability, rheometry, flow energy

## Abstract

Glidants and lubricants are often used to modify interparticle friction and adhesion in order to improve powder characteristics, such as flowability and compactability. Magnesium stearate (MgSt) powder is widely used as a lubricant. Shear straining causes MgSt particles to break, delaminate, and adhere to the surfaces of the host particles. In this work, a comparison is made of the effect of three mixer types on the lubricating role of MgSt particles. The flow behaviour of α-lactose monohydrate, coated with MgSt at different mass percentages of 0.2, 0.5, 1, and 5 is characterised. The mixing and coating process is carried out by dry blending using Turbula, ProCepT, and Mechanofusion. Measures have been taken to operate under equivalent mixing conditions, as reported in the literature. The flow resistance of the coated samples is measured using the FT4 rheometer. The results indicate that the flow characteristics of the processed powders are remarkably similar in the cases of samples treated by Turbula and Mechanofusion, despite extreme conditions of shear strain rate. The least flow resistance of samples is observed in the case of samples treated by the ProCepT mixer. High-velocity collisions of particles round off the sharp corners and edges, making them less resistant to flow. The optimal percentage of magnesium stearate is found to be approximately 1% by weight for all mixer types, as the addition of higher amounts of lubricant does not further improve the flowability of the material.

## 1. Introduction

Lubricants and glidants are commonly used in powder processing to reduce bulk powder friction in order to promote flowability and impart other desirable attributes, such as tabletability and compactability [1,2,3]. Good examples are magnesium stearate (MgSt) acting as a lubricant [4,5], and nano-particles of silica acting as a glidant [6,7]. Their use is highly desirable for preparing appropriate formulations for further processing, such as compaction and tabletting in the pharmaceutical industry [8]. The coating process is carried out by mixing, for which a wide variety of mixers are available [9]. By adding a small quantity of lubricant/glidant to a powder system, the energetic sites on the host particle surfaces get separated by a thin layer of the lubricant, thereby reducing the van der Waals interactions and sliding friction significantly [10], thus, the term ‘flow aid’ is used to describe their functionality. Due to the huge size difference between the host and guest particles, the strong intermolecular forces between them cause the guest particles to adhere strongly to the surfaces of the host particles. Moreover, shear straining in the presence of lubricants and glidants modifies the surface characteristics of the host powder, and produces new functionalities and features [11,12]. The quantity of flow aids that is necessary to achieve an optimal flow performance remains unknown to date. Conesa et al. [13] found that at 0.3 wt%, silica-coated polyester-based particles have optimal flowability. Castellanos [14] later converted this gravimetric value into surface area coverage (SAC) and found that it was approximately 20%. The work of Fulchini et al. [15] led to the same conclusion as that of Castellanos [14]. They used the image analysis technique to quantify the SAC from SEM micrographs of zeolite-coated silanised glass beads, and found that the optimal SAC for maximum flow improvement to be approximately 20%. Beyond this optimal value, the flowabilility decreased, as the system was then dominated by guest–guest rather than host–guest particle interactions. However, the work of Jallo et al. [16], on the coating of APIs with nano-silica particles, found an optimal SAC value of 293% instead, implying a multilayer coating gives rise to better flow performance. Sato et al. [17] coated sugar granules with MgSt powder in a Cyclomix mixer and reported that the guest particles were initially covered discretely, and then formed a film over the surfaces of the host particles. In addition, attrition was induced by excessive operating conditions, which resulted in the worsening of dry coating performance.

Through coating α-lactose monohydrate (α-LM) crystals with MgSt using the Mechanofusion device, Zhou et al. [18] found that the optimum SAC to be 64.5%, estimated from X-ray photoelectron spectroscopy and time-of-flight of the secondary ion mass spectrometry (Tof-SIMS). However, MgSt delaminates upon shearing [19,20,21] and the optimal amount would depend on the mixer type and shearing conditions. Moreover, Hussain et al. [19] propose that a Langmuir-type coating is first achieved with MgSt, followed by a patchy coverage in powder form. Therefore, the characterisation of SAC of MgSt on lactose crystals has yet to be satisfactorily established.

The preparation of a powder formulation is usually carried out on a small scale in a laboratory mixer, and extension to a large-scale operation is very challenging. Therefore, a ‘recipe’ for mixing equivalency is highly desirable. The attention here is on batch mixing, for which the literature is vast, addressing numerous aspects. These include the evaluation of the performance of various mixer types, assessment of mixing time and operational scale, operating conditions, the optimal amount of flow aids, and the development of models [5,12,22,23]. Horibe et al. [24] recently assessed the effect of mixing time and the concentration of flow aids on the flow properties of the end product. They found that the mixing time has a higher correlation to the flow properties than the concentration of flow aids, signifying the importance of the process and mixing conditions in improving the flow behaviour of a particulate system. As quoted from Bridgwater’s review paper [9], process design and operation are largely based on judgement rather than science. Finding the optimal mixing conditions for new materials is often a time-consuming and challenging task. The quality of a mixture depends on the mixer selected. Asachi et al. [25] critically evaluated current techniques for the evaluation of powder mixing. Of special interest here is the work of Barling et al. [26] who carried out an experimental evaluation of various mixer types, and presented a methodology for establishing an ‘equivalent extent’ of batch mixing amongst various mixer types at different scales. They used fine iron oxide powder as a tracer, and evaluated its spreading over the surfaces of α-lactose monohydrate in several batch mixing devices by monitoring the changes in the colour intensity and hue through a colour measurement methodology. The powder blend changed colour as a result of mixing and coating. By quantifying changes in the intensity and hue of the colour of the mixture, the extent of mixing and coating of iron oxide was determined, based on which a mixing ‘equivalency’ was proposed for different mixer types. It was found that under specific operating conditions, a mixing equivalency could be established among different coating devices, namely Turbula T2F (figure-eight tumbler type), Key International KG5 (high shear mixer, two sizes, 1 L and 5 L), Hosokawa AMS-MINI Mechanofusion (extreme shear rate), T.K. Fielder high shear mixers (TRV25 and PMA65), and Diosna P100 (high shear mixer type).

In this work, the above equivalency criterion is followed and applied to the coating of α-LM by MgSt powder. In particular, samples treated by different mixers, but with equivalent mixing conditions, are prepared and analysed for the effect of MgSt on the flow behaviour of α-LM. Three types of mixers, Turbula, Mechanofusion, and ProCepT high shear mixer are used. The last one is close in geometry and operation to the T.K. Fielder TRV25, 1 L high shear mixer. The equivalent mixing conditions, estimated based on the work of Barling et al. [26], are given in Table 1. The flowability of the coated samples, prepared at different MgSt compositions and mixer types, is then evaluated based on the mechanical work expended to penetrate a rotating impeller into a confined powder bed using the Freeman Technology FT4 rheometer. This will also enable the sensitivity to the shear strain rate to be quantified. The outcome provides a guideline on the amount of MgSt to be used for its effect on the flowability.

## 2. Materials and Methods

### 2.1. α-Lactose Monohydrate (α-LM) Crystals

α-lactose monohydrate (C_12_H_22_C_11_·H_2_O) is the hydrate crystal form of lactose sugar, widely used in the pharmaceutical industry as an excipient for tablet formulation as well as a host for the delivery of the active pharmaceutical ingredient (API) in the dry powder inhaler. The α-LM crystals used in this work were ‘Pharmatose 50M’ donated by DFE Pharma. They were first classified by sieving into the size range from 200 to 400 μm using BS410 sieves. Large particles were used deliberately so that the fluid drag of the medium does not affect the flowability testing. The surfaces of α-LM are very energetic with heterogeneous energy distribution, referred to as hot spots [27]. They attract a considerable amount of very fine particles of the same material, due to attrition during manufacturing, herein referred to as debris [28]. In fact, for dry powder inhalation, additional fine α-LM powder is often added to blind these hot spots, so that the adhesion and detachment of the active pharmaceutical ingredients are more controllable [29]. The crystals were therefore washed and rinsed in a fine sieve to separate the adhered lactose debris with isopropyl alcohol (propa-2-ol, supplied by VWR, Leighton Buzzard, UK). Crystals of α-LM are not soluble in this liquid carrier, and the process removes fines that are adhered to the crystal surfaces [30]. Following this stage, the crystals were left in a fume hood to air dry at room temperature prior to further analysis.

### 2.2. Magnesium Stearate (MgSt)

Magnesium stearate, a solid soap with a chemical composition of Mg (C_18_H_35_O_2_)_2_, is a white cohesive powder, commonly used as a lubricant in tablet and capsule formulations to reduce friction, and to a lesser extent as a glidant and anti-adherent. In the latter case, it is also used in dry powder inhaler formulations. Pure MgSt is often not available commercially, as it is very cohesive and does not possess the best lubricating properties [20]. Commercial MgSt powder is usually a blend of several different fatty acids (for example stearic acid and palmitic acid), which performs better as a lubricant compared to its unblended counterpart. The MgSt powder (MF-2-V Premium grade, vegetable source) used in this work was donated by Peter Greven (Bad Münstereifel, Germany). It has stearic acid and palmitic acid contents of between 63% and 27%, respectively. It is in crystalline form and has plate-shaped morphology. It is easily deformed and smeared over surfaces by frictional traction, so its size changes during the mixing.

### 2.3. Coating α-Lactose Monohydrate Crystals with Magnesium Stearate Powder

The washed and dried α-LM crystals were divided into a series of smaller samples, each having a fixed mass of 30 g. These samples were subsequently subjected to blending with MgSt powder of different weight percentages (0.2, 0.5, 1, 2, and 5% *w*/*w*) through mechanical means using a Turbula T2F mixer (GlenMills, Clifton, NJ, USA), high shear granulator (ProCepT, Zelzate, Belgium), and Mechanofusion mixer (Hosokawa Micron Ltd., Cheshire, UK). The operating conditions used for these mixing and coating devices (Table 1) were selected according to the ‘mixing equivalency’ procedure proposed by Barling et al. [26], assuming that the ProCepT design and operation are similar to those of the TRV25 mixer.

Blending of the α-LM and MgSt powder mixtures in the Turbula mixer was performed through a three-dimensional harmonic interaction of rotation, translation, and inversion of a 1 L glass vessel, in which they were tumbled around and mixed. The second mixer was the ProCepT high shear granulator. It has a three-bladed impeller in a 250 mL glass vessel. In the Mechanofusion device, the powder is pressed against the wall by centrifugal action and sheared against a press head, by which MgSt is ‘smeared’ over the surfaces of α-LM.

### 2.4. Flowability Measurement using FT4 Rheometer

The influence of MgSt coating on the flowability of α-LM particles was assessed using an FT4 Rheometer (Freeman Technology, Tewkesbury, UK). This is a dynamic powder tester, which measures the mechanical work (commonly referred to as the ‘flow energy’) expended by a two-bladed impeller, as it rotates and penetrates through a bed of powder of known volume in a glass vessel. In the downward motion, the impeller rotates anti-clockwise and applies compressive and shear stresses on the powder bed. Expressing the work per unit mass of the powder, which has undergone shear straining, is termed the specific downward flow energy (SDFE). It is indicative of the ease with which bulk powder flows under compressive and shear stresses. Moreover, as the impeller is retracted from the base to the free surface, whilst cutting and lifting the bed, the associated work per unit mass of the sheared bed is commonly referred to as the specific upward flow energy (SUFE). Under this configuration, the powder bed is relatively unconfined and is subjected to tensile stresses, hence the energy expenditure is highly dependent on the bulk cohesion. Bulk friction and cohesion play a role here, but the former is the more dominant contributor in the downward test, as it will be shown in the difference of the results between SDFE and SUFE later below. Samples of α-LM crystals were treated with five different mass percentages of MgSt (0.2, 0.5, 1.0, and 5.0%) using the three mixing technologies described above. Triplicate measurements of both specific downward and upward flow energies were carried out and the results are reported below.

### 2.5. Powder Stability and Sensitivity to Shear Strain Rate

**Figure 1:**
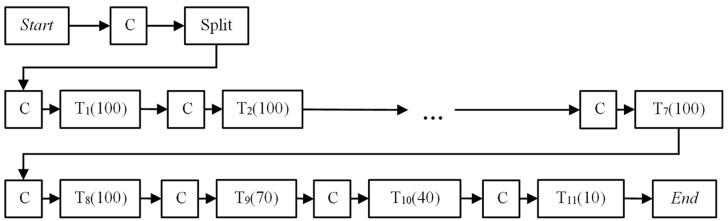
Flow chart of 11 standard FT4 tests (C: Conditioning; T*_N_*(RPM)): Test number *N* at specific RPM).

Powder beds may undergo segregation and attrition under shear straining, depending on the mechanical strength and particle size distribution. By repeating the measurement of powder flow energy in a series of consecutive tests a number of times, and comparing the energy recorded for each repetition, the stability of a powder can be inferred. The measurement can also be extended to assess the shear strain rate sensitivity of a powder by varying the impeller speed. A “stable” and “strain rate insensitive” powder should result in little to no change in the flow energy regardless of the number of times the measurement is repeated or different impeller speeds used. The standard procedure, as recommended by Freeman Technology, is followed here [31]. It involves repeating the flow energy measurement of the same sample at an impeller speed of 100 RPM eight times (referred to Test 1 to 8), followed by another three measurements (Test 8 to 11) at decreasing impeller speeds of 70, 40, and 10 RPM, as shown in Figure 1. A conditioning cycle is run between two consecutive measurements to remove any residual strains and stresses in the powder from the previous measurement.

### 2.6. Quantification of Particle Breakage through Sieving

Depending on the stresses experienced, the relative motion of particles, with respect to each other, could cause surface wear, attrition, or even fragmentation if the particles are weak. As the α-LM crystals are subjected to different levels of mechanical stresses during the blending process, a certain extent of damage is inflicted. Size classification by sieving of crystals, treated with the three mixing technologies discussed earlier, indicates their breakage of the crystals. In addition to the sieve corresponding to the lower limit of the sieve cuts used to initially classify the crystals (200 μm), two smaller sieve sizes (180 and 150 μm) were also used to analyse the debris formed. The size analysis was repeated three times for each of the mixing technologies.

## 3. Experimental Results and Discussions

### 3.1. Effect of Washing on the Flow Behaviour of α-LM

**Figure 2:**
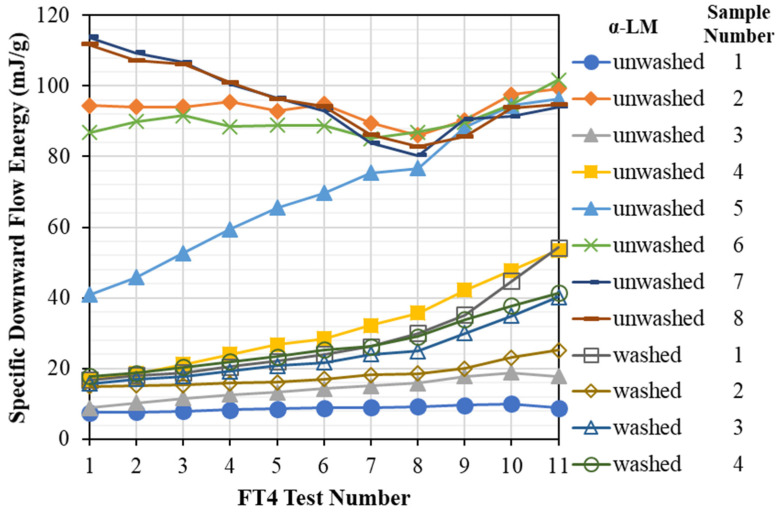
Specific downward flow energy of α-lactose monohydrate (α-LM) for twelve samples, eight samples as received and unwashed, and four samples washed with isopropyl alcohol, showing sample to sample variation. Data are for eleven consecutively repeated tests for each sample, showing test stability and strain rate sensitivity, the first eight at 100 RPM and the last three at 70, 40, and 10 RPM.

The presence of fines in a granular assembly changes its flow properties (see for example [32]). The consequences of having fines in a particulate system could be double-edged. In some cases, fines could provide a gliding effect by three-body rolling [15]. Alternatively, the presence of fines could result in a more compact and cohesive packing of the powder bed, leading to poor flow [33,34]. A comparison of the specific downward flow energy (SDFE) measured for twelve samples is shown in Figure 2; eight samples as supplied, i.e., unwashed, and four washed samples, showing test to test variation. For each sample, eleven consecutive tests were carried out to test their stability and strain rate sensitivity, with the first eight tests conducted at 100 RPM and the rest, i.e., nine to eleven were run at decreasing impeller speeds of 70, 40, and 10 RPM. The SDFE recorded for the unwashed α-LM samples (filled symbols) varies widely. The data recorded in Test 1 (*cf*. Figure 1) for the fresh samples are taken as the most representative of all the tests. The powder bed carries no previous measurement history in this case and has not experienced attrition. Despite this, the SDFE of the eight unwashed samples measured in Test 1 still ranges from approximately 9 mJ/g to ~120 mJ/g, an approximately 12-fold difference. In addition, the observed trend also varies greatly when the test is repeated (*cf*. Sample 5). This shows that the presence of fines in α-LM samples has an adverse effect on the stability of the powder flow in terms of temporal repeatability of the test results. This could be attributed to the detachment of the debris and its segregation. The strain rate sensitivity tests (Tests 8 to 11) show a fairly consistent upward trend of the SDFE as the impeller speed is reduced from 100 to 10 RPM. Compared to the unwashed samples, washed α-LM samples (empty symbols) exhibit a more repeatable pattern. Using the same sample, the resistance of the powder bed to flow is found to only increase marginally from Tests 1 to8. Decreasing the impeller speed (Tests 9 to 11), the SDFE increases, similar to the trend of the unwashed samples. In general, the SDFE recorded for the washed samples is much lower than that of the unwashed ones, indicating better flowability.

### 3.2. Effect of MgSt on α-LM Bulk Flow

**Figure 3:**
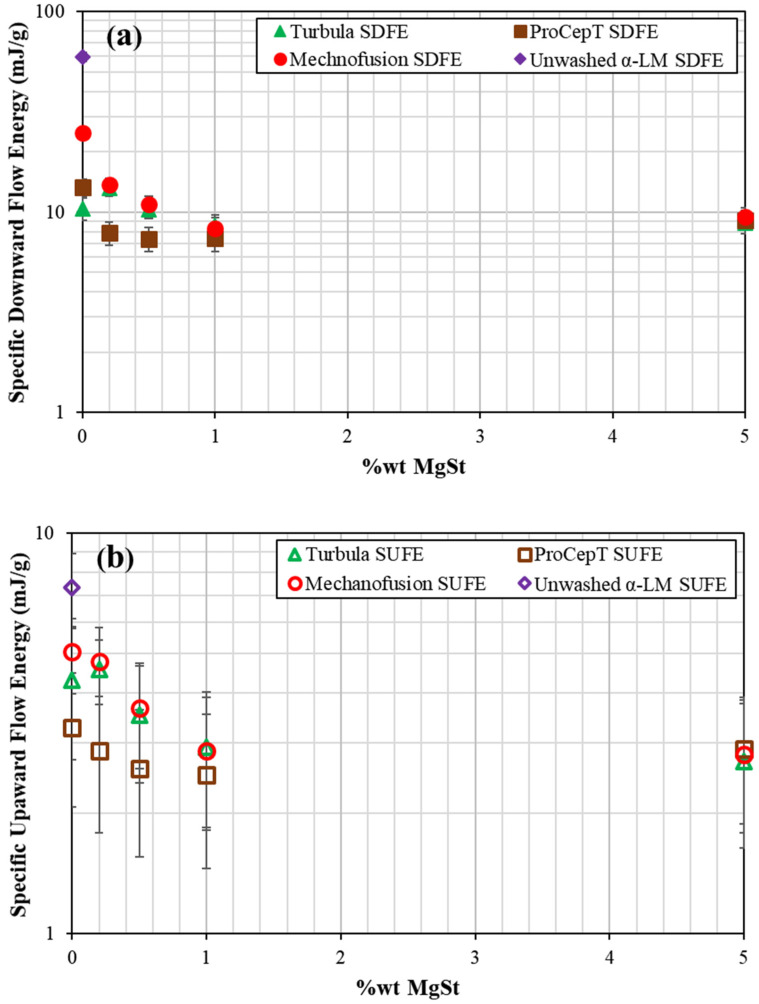
Specific flow energies of α-lactose monohydrate treated with different percentages of magnesium stearate in the three mixers. (**a**) Downward test; (**b**) Upward test. Each data point represents the mean specific flow energy of the triplicate FT4 measurements taken from Test 1. The error bar represents the standard deviation. Note: Error bars that cannot be seen are smaller than the symbols.

Coating of MgSt onto α-LM crystals changes the flow behaviour of the powder bed. However, the way coating is carried out is influential, as the interactions are no longer limited to particles of the same kind, but rather a more complex interaction of binary mixture of MgSt and α-LM. The effect of mixing MgSt with α-LM using the three mixers on the flow behaviour is shown in Figure 3. Note that each data point shown in the plot is a mean value of three measurements taken from Test 1 for every MgSt composition tested, and the associated error bar represents the standard deviation of the three repetitions.

The diamond data point legends 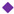 and 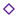 in Figure 3a,b represent the specific downward and upward flow energies, respectively, recorded for unwashed and untreated α-LM samples (mixing done without the addition of α-LM). With the addition of MgSt, the ProCepT-treated samples exhibit the least resistance to flow. Samples treated by Turbula and Mechanofusion exhibit remarkably similar specific flow energy for all MgSt concentrations, despite having undergone extreme shear strain rates. For all mixer types, increasing the composition of MgSt to 1 wt% improves the flow performance. Above 1 wt%, the specific upward and downward flow energies both converge to asymptotic levels, suggesting that the flow behaviour of the α-LM samples has now stabilised and is dominated by MgSt. Notably, the concentration of 1 wt% MgSt appears to be the threshold value, beyond which there is no significant improvement in the flow performance of α-LM, regardless of the type of mixer used.

An interesting phenomenon is observed in the strain rate sensitivity of the α-LM samples when MgSt is added. For α-LM samples alone, reducing the impeller speed leads to an increase in the specific flow energies (Tests 8–11 in Figure 2). The opposite trend prevails for the samples coated with MgSt for all three cases of mixer type, as shown in Supplementary Material, Figures S1–S3.

### 3.3. Effect of Mixer Type on Powder Flow

**Figure 4:**
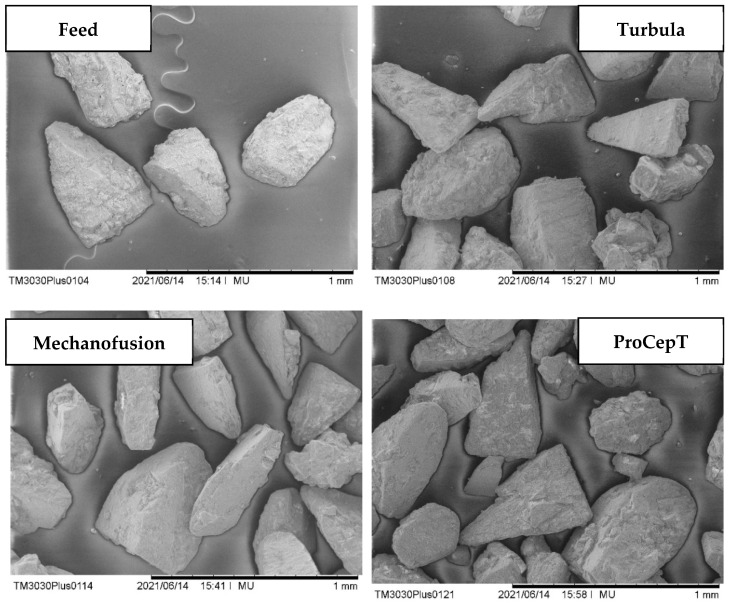
Scanning electron microscope images of isopropyl-alcohol-washed α-lactose monohydrate crystals treated with Turbula, Mechanofusion, and ProCepT with 0.2 wt% MgSt, showing notable breakage in the case of ProCepT mixer.

The operating procedure used for mixing α-LM and MgSt follows the work of Barling et al. [26]. According to their work, a mixing equivalency is achieved amongst the three mixers when the mixing is done under the operating conditions specified in Table 1. A comparison of the SDFE and SUFE recorded for the three mixers at different mass percentages of MgSt is shown in Figure 3a,b, respectively. There exists a large difference in the SDFE among the three mixers at 0% MgSt. The α-LM samples treated with Mechanofusion have the highest SDFE (~25 mJ/g) and those treated with Turbula have the lowest SDFE (~10 mJ/g). The difference among the three mixers diminishes with the MgSt wt% and converges at 1 wt% MgSt. Apart from 0% MgSt, samples treated with Turbula and Mechanofusion show almost identical behaviour, suggesting a mixing equivalency is achieved using the proposed operating procedure. Unfortunately, the same remark could not be made for ProCepT-treated samples. In this case, the difference in SDFE between the initial two consecutive cases (0 and 0.1 wt% MgSt) diminishes quickly, and then reaches a plateau. The results seem to suggest that ProCepT is a more efficient coating device compared to Turbula and Mechanofusion since a lower amount of MgSt is needed to improve the flow behaviour of the α-LM bulk solids. However, the ‘improved’ flowability seen in ProCepT at low MgSt wt% is likely due to the rounding of the corners and edges of the crystals, rather than the efficiency of the mixer in spreading MgSt over the surfaces of α-LM crystals. The latter is most effective when a dense particle bed is strongly sheared. This facilitates the ‘smearing’ of MgSt particles over the surfaces of the host particles. In the case of the ProCepT mixer, the impeller action aerates the bed, thereby reducing the bulk friction and shear viscosity of the particle bed. The scanning electron microscope images, taken from a Hitachi Benchtop SEM TM3030 Plus, as shown in Figure 4, indicate that chips and fragments are present in ProCepT-treated samples, and, also, to a lesser extent in the case of Mechanofusion, as it can be implicitly inferred from the extent of breakage reported below. The magnification of the images is shown as a line bar with a 1 mm scale.

In terms of SUFE, the trend of the results of samples treated with MgSt by Turbula and Mechanofusion almost overlap. ProCepT-treated samples generally have much better flow performance compared to Turbula- and Mechanofusion-treated samples at the same MgSt wt%.

### 3.4. Extent of Breakage

α-LM crystals experience different levels of mechanical stresses due to agitation in the three mixers. Vigorous relative motion between the particles during the mixing process damages the particles, and a reduction in particle size is expected. Following the mixing process, a sieving analysis is performed to quantify and compare the extent of size reduction of the α-LM particles in the three mixers. The size distributions of the α-LM samples treated with Turbula, ProCepT, and Mechanofusion with 0.2 wt% MgSt are shown in Table 2. The size of the α-LM particles is reduced as a result of mixing, but only to a small extent. The following criterion is adopted to quantify the extent of breakage: the mass of particles collected by a sieve that is two sizes (according to BS410 sieves) below the feed sieve size is used as a breakage indicator. The extent of particle breakage is found to be in the range of ~0.1%. This suggests that the breakage mode of the α-LM particles in the three mixers of interest is dominated by attrition and surface abrasion. The amount of debris collected in Turbula-treated samples is the least amongst the three mixers. This is expected as the mixing action is the gentlest amongst the three mixers and involves no moving mechanical part that comes into direct contact with the particles in the mixing chamber.

## 4. Conclusions

The influence of mixer type on the coating of α-LM crystals with a small quantity of MgSt powder has been assessed. Three different types of mixers, i.e., Turbula, ProCepT, and Mechanofusion have been used. The assessment method is based on the effect of MgSt coating on flowability, as measured by the mechanical work expended by penetrating a rotating impeller into a powder bed by using an FT4 rheometer. The flowability of α-LM crystals is affected by the presence of debris, i.e., fine particles of the same material, adhered to their surfaces, causing a wide sample to sample variation. Washing the α-LM crystals using a liquid carrier, which does not dissolve them, reduces the variation as well as the magnitude of the expended work significantly, implying improved flowability. The addition of MgSt to α-LM particles further improves the flowability for powders coated in all of the three mixers under equivalent mixing conditions, described in the work of Barling et al. [26]. There exists a threshold at ~1 wt% MgSt above which the further addition of MgSt does not improve the bulk flow performance of α-LM. Samples coated by both Turbula and Mechanofusion mixers show remarkably similar flow performance, despite extreme shear strain rate. At the same MgSt composition, the ProCepT-treated α-LM samples consistently perform better in terms of flow behaviour. This is attributed to collisional impacts in this mixer, rounding off sharp corners and edges. Mixing equivalency is best achieved between the Turbula and Mechanofusion mixers.

A breakage analysis due to mixing is also performed, and the results suggest that α-LM samples coated using Turbula experience the least damage. The particle size distribution of the treated samples reveals that attrition and surface abrasion create the dominant breakage mode, particularly in the case of Turbula and Mechanofusion mixers, with their breakage extent being found to be in the range of approximately 0.1%.

## Figures and Tables

**Table 1:** Mixing speed and time for achieving mixing equivalency adapted from Barling et al. [26], Elsevier, 2015.

Coating Device	Speed (RPM)	Mixing Time (Minutes)
Turbula	72	45
TRV25	235	20
Mechanofusion	600	1

**Table 2:** Particle size distribution of α-lactose monohydrate particles, treated by Turbula, ProCepT, and Mechanofusion with 0.2 wt% MgSt under equivalent mixing conditions according to Barling et al. [26].

Sieve Size, µm	% Mass					
	Mechanofusion		ProCepT		Turbula	
	Mean	SD	Mean	SD	Mean	SD
<150	0.13	0.03	0.19	0.10	0.08	0.05
150–180	0.18	0.18	0.15	0.00	0.19	0.11
180–200	0.62	0.33	1.11	0.24	0.41	0.15
>200	99.06	0.29	98.55	0.29	99.32	0.23

## Supplementary Materials

The following are available online at https://www.mdpi.com/article/10.3390/pharmaceutics13081211/s1, Figure S1. Specific downward flow energy of α-lactose monohydrate coated with different mass percentages of magnesium stearate, prepared by Turbula, for 11 consecutively-repeated tests for each sample. Figure S2. Specific downward flow energy of α-lactose monohydrate coated with different mass percentages of magnesium stearate, prepared by ProCepT, for 11 consecutively-repeated tests for each sample. Figure S3. Specific downward flow energy of α-lactose monohydrate coated with different mass percentages of magnesium stearate, prepared by Mechanofusion, for 11 consecutively-repeated tests for each sample.
