# Supplementary material for: Effect of Mixer Type on Particle Coating by Magnesium Stearate for Friction and Adhesion Modification
Source: Pharmaceutics. 2021 Aug 5;13(8):1211. doi: 10.3390/pharmaceutics13081211 (PMC8400245; doi:10.3390/pharmaceutics13081211)
Supplement: Supplementary file 1 [file pharmaceutics-13-01211-s001.zip › pharmaceutics-1324021-supplementary.pdf]

pharmaceutics

Supplementary Materials: Effect of Mixer Type on Particle Coating by Magnesium Stearate for Friction and Adhesion Modification

Wei Pin Goh, Ana Montoya Sanavia and Mojtaba Ghadiri

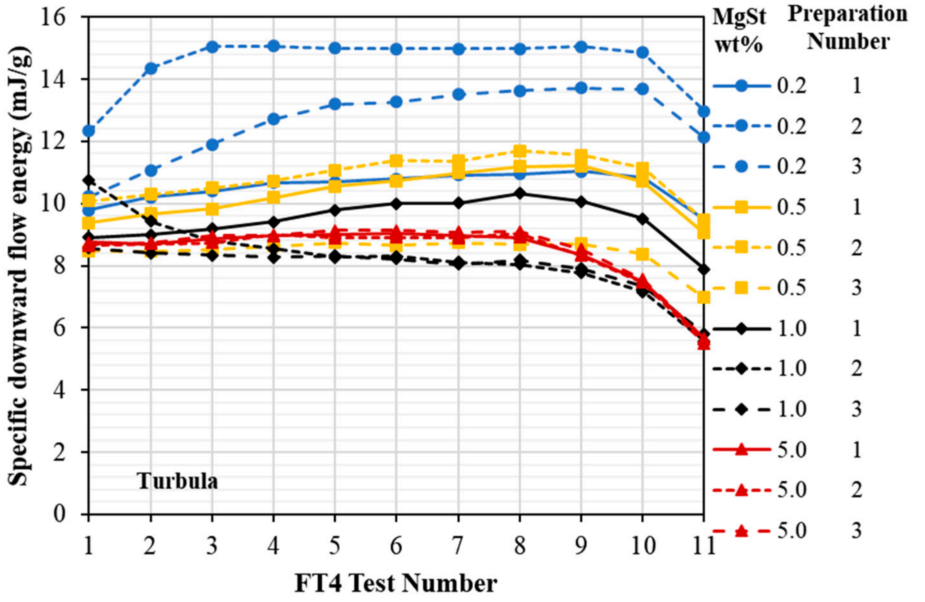

Figure S1. Specific downward flow energy of α-lactose monohydrate coated with different mass percentages of magnesium stearate, prepared by Turbula, for 11 consecutively-repeated tests for each sample. The results show sample variation, stability and strain rate sensitivity. The first eight tests are at 100 RPM and the last three at 70, 40, 10 RPM.

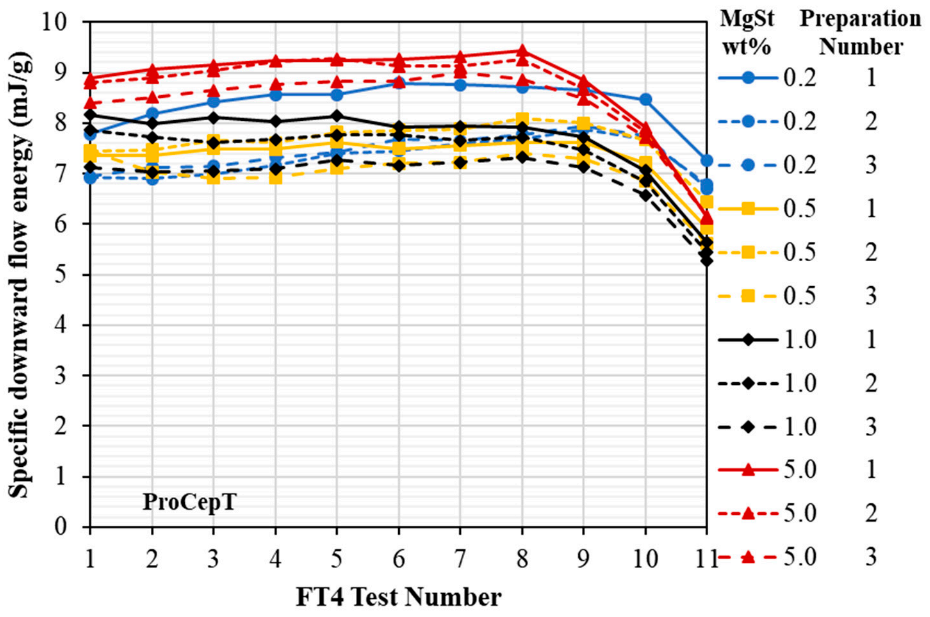

Figure S2. Specific downward flow energy of α-lactose monohydrate coated with different mass percentages of magnesium stearate, prepared by ProCepT, for 11 consecutively-repeated tests for

each sample. The results show sample variation, stability and strain rate sensitivity. The first eight tests are at 100 RPM and the last three at 70, 40, 10 RPM.

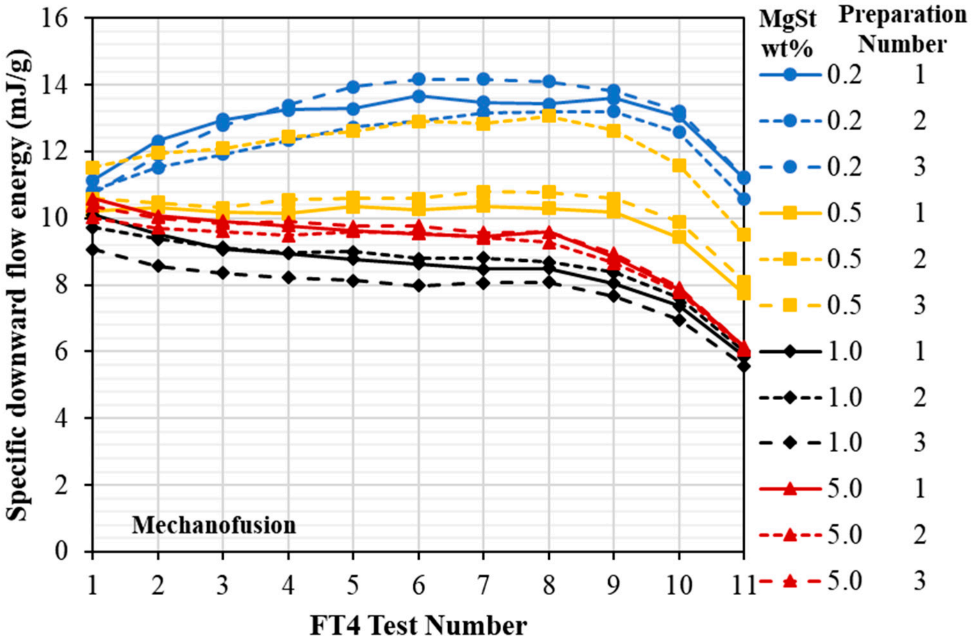

Figure S3. Specific downward flow energy of α-lactose monohydrate coated with different mass percentages of magnesium stearate, prepared by Mechanofusion, for 11 consecutively-repeated tests for each sample. The results show sample variation, stability and strain rate sensitivity. The first eight tests are at 100 RPM and the last three at 70, 40, 10 RPM.
